# Comparing Antemortem CT–Angiography Data with Autopsy Findings in Regard to Anterior Communicating Artery Aneurysms

**DOI:** 10.3390/neurosci6030081

**Published:** 2025-08-18

**Authors:** Ana Maria Dumitrescu, Dragos Andrei Chiran, Cristinel Ionel Stan, Cringuta Mariana Paraschiv, Nicolaie Dobrin, Alexandru Chiriac, Maria Magdalena Leon, Lucia Corina Dima-Cozma, Cristina Gena Dascalu, Ana Marina Radulescu, Roxana Florentina Gavril, Anca Sava

**Affiliations:** 1 Department of Morpho-Functional Sciences I, “Grigore T. Popa” University of Medicine and Pharmacy, 700115 Iasi, Romania; ana-maria_dumitrescu@umfiasi.ro (A.M.D.); cristinel.stan@umfiasi.ro (C.I.S.); ana.marina.radulescu@umfiasi.ro (A.M.R.); roxana-florentina.sufaru@umfiasi.ro (R.F.G.); sava.anca@umfiasi.ro (A.S.); 2 Department of Medical Sciences I, “Grigore T. Popa” University of Medicine and Pharmacy, 700115 Iasi, Romania; cringuta.paraschiv@umfiasi.ro (C.M.P.); maria.leon@umfiasi.ro (M.M.L.); cozma.dima@umfiasi.ro (L.C.D.-C.); 3 Internal Medicine Clinic, Clinical CF Hospital, 700506 Iasi, Romania; 4 Neurointerventional Surgery Department, “Prof. Dr. N. Oblu” Emergency Clinical Hospital, 700309 Iasi, Romania; dobrin_nicolaie@yahoo.com (N.D.); alexandru.chiriac@umfiasi.ro (A.C.); 5 Department of Surgery II, “Grigore T. Popa” University of Medicine and Pharmacy, 700115 Iasi, Romania; 6 Department of Medical Informatics and Biostatistics, “Grigore T. Popa” University of Medicine and Pharmacy, 700115 Iasi, Romania; cristina.dascalu@umfiasi.ro; 7 Department of Pathology, “Prof. Dr. N. Oblu” Emergency Clinical Hospital, 700309 Iasi, Romania

**Keywords:** anterior communicating artery aneurysm, autopsy, computed tomography angiography, 3D reconstruction, anatomical variant, circle of Willis

## Abstract

Background: The literature shows that anterior communicating artery (AcoA) aneurysms are the most common intracranial aneurysms. To date, there has only been one postmortem study focused on the correlations between autopsy findings and imaging results in cases of intracranial aneurysms associated with anatomical variants of the circle of Willis (CW). Methods: We investigated the anatomical variants of the CW associated with the occurrence and rupture of AcoA aneurysms by performing comparative analyses, in the same patients, of postmortem autopsy data with antemortem computed tomography–angiography (CTA) results obtained in the first 48 h after the onset of subarachnoid hemorrhage. Our retrospective observational study identified the anatomical variants of the CW at autopsy in 16 deceased adult Romanian patients with AcoA aneurysms over a 12-year period (2010–2022). Results: The autopsy findings revealed that the AcoA ruptured aneurysms had a mean external diameter of 9.50 mm, and 71.4% of them presented three or four anatomical variants inside the same CW. The initial antemortem CTA examination correctly located the AcoA aneurysms in all cases (100%), and an anatomical variant of the CW was only noted in 18.75% of patients. The final postmortem re-analyzed the same CTA images identified in all cases (100%), focusing on both the AcoA aneurysm and all anatomical variants of the CW found during the autopsies. Conclusions: Although it was previously thought that the occurrence of AcoA aneurysms is related only to the hemodynamic changes induced by the nearby arterial anatomical variants, we identified the simultaneous involvement of at least one hypoplastic artery and one or two PCA fetal-type anatomical variants that were located in both the anterior and posterior parts of the CW. Furthermore, if sufficient time is devoted to the CT–angiography analysis and interpretation of the images, anatomical variants of the circle of Willis associated with AcoA aneurysms can be identified as accurately as they are in invasive postmortem autopsy examinations.

## 1. Introduction

Anterior communicating artery (AcoA) aneurysms are the most common intracranial aneurysms, accounting for 23–40% of all cases [1,2], and have the highest risk of rupture [3], thus representing an important vascular pathology in neurosurgical clinics. In a previous autopsy study [4], we identified 13.13% of intracranial aneurysms in a cohort of 221 patients that died due to neurological and neurosurgical diseases in a tertiary hospital. AcoA aneurysms represented a much higher percentage (55.2%) of intracranial aneurysms than reported in the literature.

As far as we know, there are only two studies [5,6] which have considered the possible correlations between imaging results and autopsy findings in the case of anatomical variants of the circle of Willis (CW), but only one of them analyzed possible correlations between the development of intracranial aneurysms and the presence of anatomical variants of the circle of Willis.

One of these two studies [5], conducted half a century ago, explores the use of postmortem cerebral angiography in the identification of intracranial aneurysms. The authors describe the technique of injecting contrast medium into the cerebral vasculature after death and present the advantages of this method with regard to identifying vascular lesions. The analysis was performed on a significant number of cases (291 brains) and highlighted the fact that postmortem angiography is a valuable method to complement autopsy, providing objective evidence of cerebral vascular pathology.

In the second study [6], which was conducted a decade ago, the authors examined 250 circles of Willis obtained using forensic autopsy and 250 circles of Willis from patients undergoing computed tomography–angiography in order to evaluate, by comparison, the frequency of anatomical variants at the level of these anatomical structures. They found that anatomical variants of the CW were identified in almost 60% of patients for both groups and concluded that there was no statistically significant difference in the overall frequency of circle variations between these two types of examinations.

Correlation of the antemortem CTA findings with postmortem autopsy data regarding AcoA aneurysm’s association with anatomical variants of circle of Willis in the same group of patients has not yet been achieved either in Romania or in other countries. The present study aims to investigate the significance of anatomical variants of the CW with regard to the occurrence and rupture of AcoA aneurysms via comparative analysis of the data obtained at autopsy with the results provided by CTA examination performed antemortem in the first 48 h after the onset of subarachnoid hemorrhage. It uses the same population group, consisting of Romanian adult patients with ruptured and unruptured AcoA aneurysms.

## 2. Materials and Methods

The present research was approved by the Ethics Committee of the “Prof. Dr. N. Oblu” Emergency Clinical Hospital in Iași, Romania (No. 7465/8 May 2020), and by the Research Ethics Commission of the “Grigore T. Popa” University of Medicine and Pharmacy, Iași, Romania (No. 192/31 May 2022).

Upon admission to the “Prof. Dr. N. Oblu” Emergency Clinical Hospital in Iași, Romania, every patient or their legal representative had to sign an Informed Consent Form for medical investigations, therapeutic procedures, participation in the medical educational process, and compliance with internal regulations for hospitalized patients. In doing so, each patient/legal representative agreed to the collection, storage, and use of the patient’s biological samples both for diagnostic purposes and for scientific research and medical instruction. The patients (or representatives on their behalf) agreed that their biological material may be photographed and published, without any other express authorization from them, as long as confidentiality is maintained.

This research was a retrospective observational descriptive study, conducted on a group of 16 adult (≥18 years old) Romanian patients with AcoA aneurysms, who were admitted to the “Prof. Dr. N. Oblu” Emergency Clinical Hospital in Iași, Romania; died during hospitalization; and then underwent autopsy in the same hospital between 1 January 2010, and 31 December 2022, at the request of the attending physician, to establish the cause of death.

The written archive of the Pathology Department of the hospital was used to collect the demographic data of those 16 patients included in this study (age and gender) and the morphological aspects identified at the level of the CW arteries [the anterior communicating artery (AcoA); the A1 precommunicating segments of the two anterior cerebral arteries (ACA), right and left, from their origins to their junctions with the AcoA; the two posterior communicating arteries (PcoA), right and left; and the P1 precommunicating segments of the two posterior cerebral arteries (PCA), right and left, from their origins to their junctions with the PcoA].

Thus, not only the type of anatomical variants of the CW arteries but also the presence and the external diameter of the AcoA aneurysm were recorded. The external diameters of the AcoA aneurysm were measured at the time of autopsy on fresh specimens, using a digital sliding caliper, with a precision of 0.1 mm.

The results of the CTA imaging performed antemortem in the emergency room, in the first 48 h from the onset of subarachnoid hemorrhage, on the same 16 patients with AcoA aneurysm, were also recorded from the archive of the Neurointerventional Surgery Department.

To visually compare the differences of the anatomical variants and the presence of AcoA aneurysms between antemortem CTA and the autopsy data of the same 16 patients, the imaging results were re-analyzed in the Interventional Angiography Laboratory of the same hospital, under the conditions in which the anatomical variants, identified at autopsy at the level of each CW with AcoA aneurysm, were already known.

Firstly, a thorough analysis of the CW anatomy and of the presence and diameters of AcoA aneurysms was performed on three-dimensional (3D) reconstruction CTA images of all 16 patients. The CTA images were obtained using the computed tomography units of the hospital that were intended for emergency diagnostic. Regarding the neurovascular studies, namely 3D CTA, a Toshiba Aquilion scanner (Toshiba Medical Systems Corporation, Tochigi, Japan) with 16 detectors was used and the images were processed using the Radiant DICOM Viewer software.

The correspondence between the 3D CTA images, analyzed both antemortem and postmortem, and the autopsy data was determined, using the latter as the standard of accuracy. These correlations were noted as follows: (1) complete agreement, if the data on the anatomical variants of the CW from 3D CTA examination corresponded entirely to those obtained during the autopsy; (2) incomplete agreement, if the data from the two studies, imaging and autopsy, partially corresponded, taking notice of the common correspondences; (3) disagreement, when imaging data on anatomical variants of the CW did not correspond to those obtained at autopsy.

Based on existing studies in the literature, which were conducted on CW obtained at autopsy, in order to assess the morphological aspects identified in the present research, we used the following definitions of the anatomical variants of the constituent arteries of the CW: (1) hypoplasia of an artery of the CW, caused by incomplete development of the corresponding vessel [7], was defined, with the exception of PcoA, by an external diameter less than 1 mm but with a patent lumen [8,9]; (2) posterior communicating artery (PcoA) hypoplasia was defined by an external diameter less than 0.5 mm, but with a patent lumen [8,9]; (3) fetal type of posterior cerebral artery (PCA) is the artery that originates from the Internal Carotid Artery (ICA) but at the same time maintains a small atretic/hypoplastic connection with the Basilar Artery (BA), and the diameter of the P1 segment of the PCA is smaller than that of the PcoA [10,11]. In this situation, the diameter of the PcoA is larger than the P1 segment of the ipsilateral PCA, which is hypoplastic [12].

For consistency with other angiographic morphological studies of the CW reported in the literature [13,14,15], the following definitions were used: (1) a hypoplastic artery was considered a vessel with an external diameter of less than 1 mm; (2) when the external diameter of the PcoA was greater than the external diameter of the P1 segment of the PCA, the anatomical variant was considered to be a PCA fetal-type artery.

Statistical analyses were performed using the SPSS Statistics software (version 29.0, Armonk, NY, USA: IBM Corp). To characterize the behavior of the data, descriptive statistics were performed: qualitative variables were characterized by calculating absolute and percentage frequencies, and quantitative variables were characterized by calculating mean± standard deviation (SD). An analytical study was carried out using the Chi-square test to investigate associations between qualitative variables. Values of the *p*-coefficient < 0.05 were considered statistically significant, and *p*-values < 0.01 were considered highly statistically significant. We also used Student’s *t*-test to test whether the difference between the response of two groups is statistically significant or not.

## 3. Results

### 3.1. Demographic Determinants for the Presence of Ruptured and Unruptured AcoA Aneurysms

In the present study, there were ten males (62.5%) and six females (37.5%), with a M:F ratio of 1.66. At the time of their death, the patients had a mean age of 62.81 years (ranging from 34 years to 86 years) (Table 1). The presence of AcoA aneurysms was associated mainly with male gender (57.1% of patients with ruptured aneurysms and 100% of patients with unruptured aneurysms were males), but there was no statistical significance for the correlation between gender and AcoA aneurysms (*p* = 0.500) (Table 1).

The age of patients (mean ± SD) with unruptured AcoA aneurysms was lower (59.50 ± 13.43 years) compared to that of patients with ruptured AcoA aneurysms (63.29 ± 12.89 years). In the group of patients with ruptured AcoA aneurysms, it was found that male patients were younger (58.50 ± 12.62 years) than female patients (69.67 ± 11.11 years). None of these data were statistically significant (*p* = 0.704) (Table 1). Most patients with AcoA aneurysms (68.8%) were older than 60 years. In the group of patients with ruptured AcoA aneurysms, most patients (71.4%) were also older than 60 years. These data were also not statistically significant (*p* = 1.000) (Table 1).

### 3.2. Correlations Between External Diameters of AcoA Aneurysms and Anatomical Variants of Circle of Willis

The mean diameter of all AcoA aneurysms taken together (ruptured and unruptured) was 8.81 mm, ranging from 4 mm to 15 mm. The mean diameter of the ruptured AcoA aneurysms was 9.50 mm, and the mean diameter of the unruptured AcoA aneurysms was 4.00 mm. These data were statistically significant (<0.001) (Table 2).

The most frequently (50.0%) ruptured AcoA aneurysms were associated with three anatomical variants located at the level of the same CW, but there were also associations with four anatomical variants (25.0%), as well as with one variant (12.5%) and two variants (12.5%) (Table 2). In total, 50.0% of all unruptured AcoA aneurysms were associated with three anatomical variants and the other 50.0% of them with four anatomical variants of the arteries constituting CW (Table 2). These data were not statistically significant (*p* = 0.767).

Ruptured AcoA aneurysms had either an external diameter of 5–9.9 mm (57.1% of all cases) or an external diameter ≥10 mm (42.9%). All unruptured AcoA aneurysms (100.0%) presented an external diameter <5 mm. However, 87.5% of all AcoA aneurysms, ruptured and unruptured, presented external diameters ≥5 mm. These data had statistical significance (*p* < 0.001) (Table 3).

In total, 64.3% of ruptured AcoA aneurysm-associated hypoplasia of at least one artery of the CW, but not a single AcoA aneurysm (0%), was associated only with PCA fetal type. However, the association of at least one hypoplastic artery of the CW with at least one PCA fetal type within the same CW was identified in 35.7% of cases with ruptured AcoA aneurysms. The unruptured AcoA aneurysm was associated either with hypoplasia of at least one artery of the CW (50.0%), or a hypoplastic variant of any artery of the CW and PCA fetal variant within the same CW (50%). We did not find any statistical significance (*p* = 1.000). Taken together, 62.5% of all AcoA aneurysms were only associated with hypoplastic anatomical variants and 37.5% of all cases were associated with at least one hypoplastic artery and a PCA fetal type, within the same CW. These results did not reveal any statistical significance (*p* = 1.000) (Table 3).

Taken together, AcoA aneurysms presented the smallest (mean ± SD) external diameter (6.50 ± 2.12 mm) when CW showed only one anatomical variant of the CW and the largest (mean ± SD) diameter (11.00 ± 4.83 mm) when CW presented four simultaneously anatomical variants. These results did not reveal any statistical significance (*p* = 0.409) (Table 4).

### 3.3. Agreement Between Autopsy Data and CT–Angiography Regarding the Presence of AcoA Aneurysm and Anatomical Variants of the Constituting Arteries of Circle of Willis at Initial Analysis and Postmortem Re-Analysis

**Figure 1:**
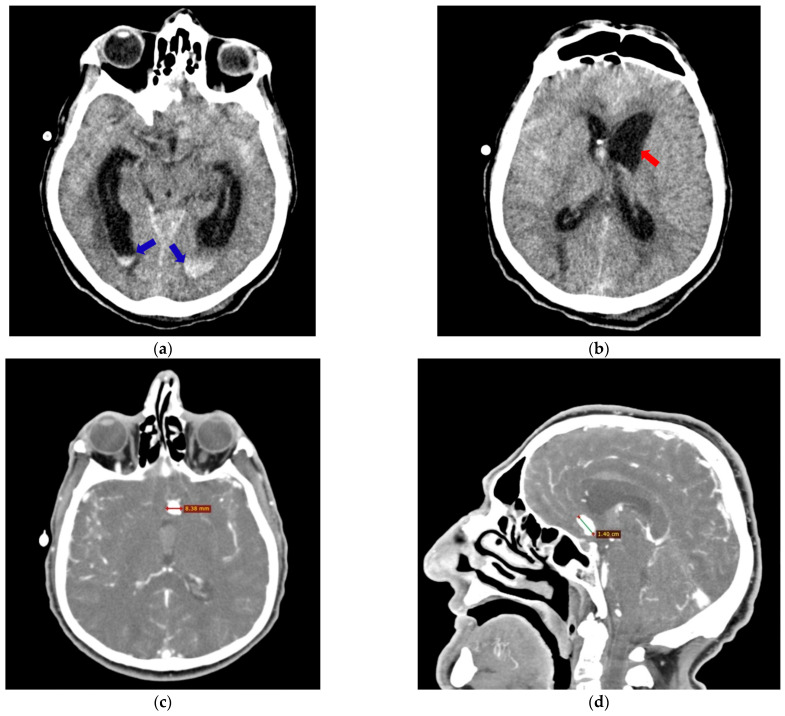
M, 63 years old. Craniocerebral CT revealed a subarachnoid hemorrhage with ventricular flooding (blue arrows), transverse section (**a**), associated with diffuse cerebral edema with obliteration of the cortical sulci and dilation of the sphenoid horns of the lateral ventricles (red arrow), transverse section (**b**), imaging features that may suggest a ruptured AcoA aneurysm. CTA identified an aneurysmal dilation of 5.9/8.38/14 mm, possibly at the level of the AcoA; transverse section (**c**) and sagittal section (**d**).

**Figure 2:**
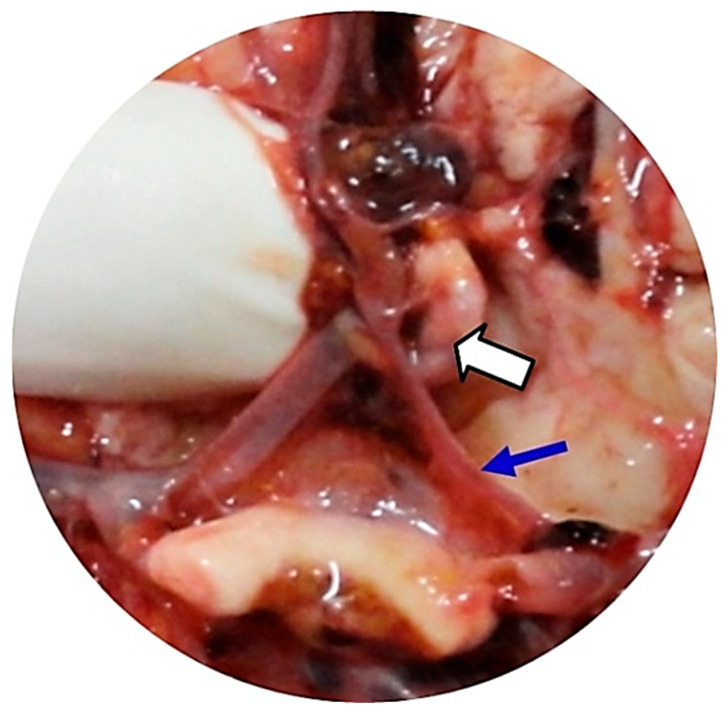
The same patient as shown in the previous figure. The autopsy demonstrated the presence of a ruptured AcoA aneurysm with 12 mm diameter (white arrow), associated with the following variants of the CW: hypoplasia of the A1 segment of left ACA (blue arrow), hypoplasia of the right PcoA, and hypoplasia of the left PcoA (not shown in the image).

**Figure 3:**
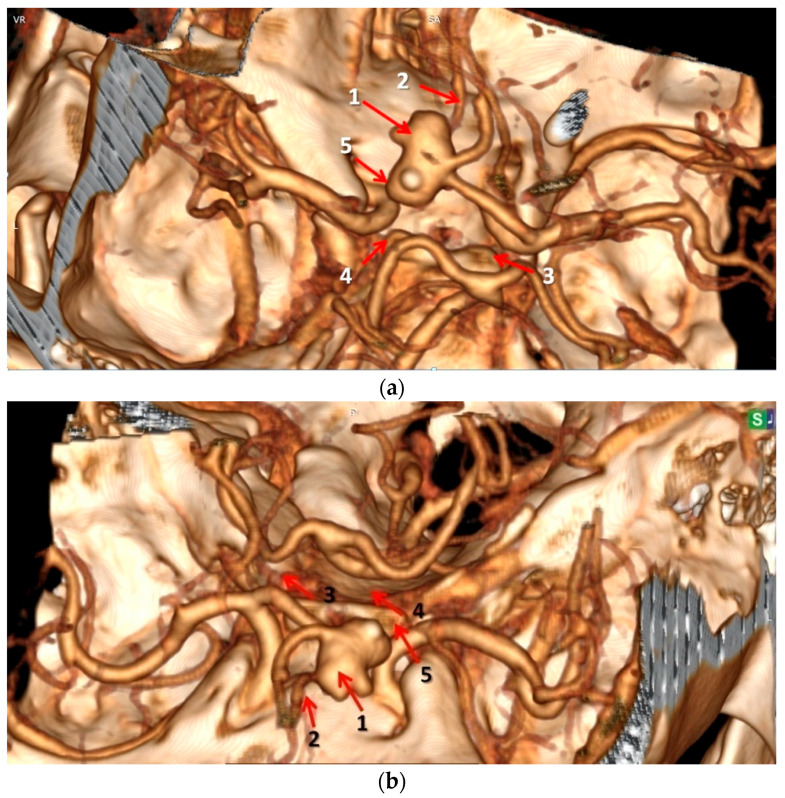
Re-examination of the three-dimensional (3D) reconstruction CTA demonstrated the following: (1) complex polylobate aneurysm of the AcoA; (2) hypoplasia of the A2 segment of the left ACA; (3) hypoplasia of the right PcoA; (4) hypoplasia of the left PcoA; (5) hypoplasia of the A1 segment of left ACA. Image at initial angle (**a**); image rotated approximately 180 degrees (**b**). VR = volume rendering, SA = superior-anterior.

Comparing the autopsy findings, during the initial, antemortem, CTA-3D scan examination, the location of the AcoA aneurysm was correctly reported, but an incomplete agreement was obtained regarding anatomical variants of the CW, as only three patients (18.8%) were found to have just hypoplasia of the A1 segment of the ACA (left or right). In 13 patients (81.3%), the presence of the AcoA aneurysm was described, but the presence of any anatomical variant of the CW was not observed. During the second, postmortem, analysis of CTA-3D scans of the brain, we correctly identified (100%) not only the AcoA aneurysms but also all the anatomical variants of the CW that were identified during the autopsy, both hypoplastic and fetal-type variants, which were located both in the anterior and posterior parts of the CW (Table 5 and Figure 1, Figure 2 and Figure 3). These data had statistical significance (*p* < 0.001).

## 4. Discussion

Imaging studies [13,16] have revealed an association between AcoA aneurysms and anatomical variants of the ACA-AcoA complex in younger patients (about 55 years old). The literature also points to a predominance of either females [13] or males [17] among patients with AcoA aneurysms. The data from the present study show that AcoA aneurysms predominantly affected male patients, with a younger mean age (about 58 years old) than female patients. However, the mean age of the group was higher than that reported in the literature. Unlike other previous studies, which were imaging-based, on living patients, our results may be a consequence of the fact that the present study involved autopsy and was realized on patients who died due to AcoA aneurysm rupture or other comorbidities, but associated with an AcoA aneurysm. It is possible that the population from the northeastern region of Romania, where these patients with AcoA aneurysms come from, presents certain particularities, as demonstrated by other authors who analyzed patients residing in the same geographical region.

One proof came from a study [18] that analyzed the role of lifestyle, associated diseases, and anatomical variants of the CW in the death of patients with AcoA aneurysm, showing that all male patients had hypertension and atherosclerotic disease, many of them being chronic alcohol users and smokers, all of these comorbidities being risk factors for patient death in the hospital. Other studies [19,20] demonstrated that cardiovascular diseases and stroke occurred in patients over 50 years of age and with metabolic syndrome, which also represent significant risk factors for death in patients with cerebrovascular pathology.

On the other hand, it is possible that the population from northeastern Romania is likely to have certain anatomical particularities at the CW level, because some recent anatomical studies identified several other anatomical variants within the population living in the same region, such as incomplete types of arcuate foramen, more prevalent on CT scans than complete types [21], and also an abnormal pneumatization of the middle nasal turbinate, also named concha bullosa, which is a congenital anomaly with a greater incidence in people living in northeastern Romania [22,23]. Moreover, there are other anatomical particularities of different structures of the brain in the population of the same geographical region [24].

Contrary to other studies [13,17], we found that AcoA aneurysms were associated with a complex constellation of simultaneous anatomical variants within the same CW, located in both the anterior and posterior parts of the CW. We identified similar findings in another study on 96 fresh brains obtained at autopsy of deceased patients with stroke and originating in the same geographical region. We noted that almost half of the cases showed anatomical variants in both the anterior and posterior parts of the CW [25].

In the present research, the mean diameter of ruptured AcoA aneurysm is more than double that of other studies, probably as a result of the association with alcohol consumption and excessive smoking because these two unhealthy habits have the known effect of further weakening the aneurismal wall [26,27,28]. Moreover, it is possible that these particular data could be correlated with the late referral of Romanian patients to a specialist, either due to the precarious socio-economic situation or the lack of medical education.

Compared to data reported by other authors [4,29,30], which showed a preponderance of the rupture of AcoA aneurysms with a mean size smaller than 5 mm, in the present postmortem research, the mean diameter of the ruptured AcoA aneurysms was significantly larger (almost 9 mm) and these data are in accordance with the International Study of Unruptured Intracranial Aneurysms [31], which stated that the anterior circulation aneurysms with a diameter ≥7 mm have a higher risk of rupture. However, the present research confirms the fact that AcoA aneurysms with diameters <7 mm may also rupture if there are some vascular risk factors [1,32], such as aneurysm location and size, previous subarachnoid hemorrhage, positive family history, smoking, heavy alcohol consumption, and arterial hypertension.

At the same time, we found that there is a relationship between aneurysm diameter, rupture risk, and the number of arteries affected by the anatomical variant. The simultaneous presence of multiple anatomical variants within the same CW was correlated with a larger diameter of the ruptured AcoA aneurysm, probably because these variants caused greater stress to the vascular wall and contributed to the formation of a larger aneurysm and to its rupture.

On the other hand, ruptured and unruptured AcoA aneurysms were mainly correlated with three or four anatomical variants of the CW, and this could indicate that the larger the number of anatomical variants, the higher the risk of developing an AcoA aneurysm. Although not statistically significant, probably due to the small number of cases, this correlation nevertheless demonstrates the significance of anatomical variants of the CW in the development of AcoA aneurysm.

There are relatively few studies demonstrating the role of anatomical variants of the CW configuration as risk factors for the development and rupture of an intracranial aneurysm [33,34,35,36]. Most studies have shown that the variability of the anterior part of the CW, in the form of a hypoplastic ACA, has an important role in the formation of AcoA aneurysms [37,38,39]. Imaging studies [3,13,40,41,42] identified ACA hypoplasia/aplasia, especially on the right side, as a risk factor contributing to the development of an AcoA aneurysm due to changes in hemodynamics in the anterior part of the CW. A recent article described a higher but non-significant association between aneurysms and incomplete CW [43].

In contrast to all these studies, the results of the present research, which used the autopsy method, identified new data according to which AcoA aneurysms are frequently associated with three or four simultaneous anatomical variants, located both in the anterior and posterior parts of the same CW. Moreover, the present study demonstrated that hypoplasia was the most frequent anatomical variant associated with AcoA aneurysm but also that one third of the cases presented an association between hypoplastic arteries with PCA fetal-type arteries in the same CW.

A recently published study confirmed our data regarding AcoA aneurysm associated with distant anatomical variants of the CW. A group of researchers from Turkey [44] demonstrated that, under the conditions of the coexistence of PCA fetal type with hypoplasia of the A1 segment of ACA, the risk of formation and rupture of an AcoA aneurysm significantly increases.

There are three theories regarding the mechanism by which AcoA aneurysm develops: genetic predisposition, congenital anomalies, or hemodynamic vascular stress [45]. Given that an AcoA aneurysm is formed within a CW with anatomical variants, often multiple, the theory of hemodynamic vascular stress is most plausible, although the contribution of genetic predisposition and a congenital anomaly of the vascular wall cannot be ruled out.

The success of endovascular treatment in intracranial aneurysms depends largely on the precise determination of their presence and location in the arterial system at the base of the brain. The accuracy of intracranial CTA is of vital importance not only in the diagnosis but also in the appropriate management of patients with intracranial aneurysms.

In the present study, the autopsy data were compared with the initial imaging data, the latter being obtained during the emergency examination in the first 48 h from the onset of subarachnoid hemorrhage. We eliminated from our study the patients with a CTA realized after this period of time as the analysis of the scans became difficult to perform because of the vasoconstriction of the arteries of the CW. However, when we compared autopsy data with these imaging data, we obtained only a small correlation as the AcoA aneurysms were correctly identified, but only anatomical variants of proximal segments of the CW were reported. Subsequently, upon thorough postmortem examination of the 3D CTA scans, when the autopsy data were already known, no difference was found between the accuracy of the CTA data and autopsy findings in terms of AcoA aneurysm identification and the type and location of all anatomical variants of the CW.

The disagreement between the CTA data and autopsy findings in the present study suggests that CTA fails to detect anatomical variants of the CW, while autopsy identifies with great precision any vascular morphology, unlike a previous study conducted by a group of Bulgarian researchers [6]. It is worth noting that the identification of some anatomical variants of the CW on 3D-CTA can be hidden by artifacts or technical limitations, such as motion artifacts, poor timing of contrast bolus, or severe vasospasm, the last obscuring hypoplastic vessels and thus leading to misinterpretation. Moreover, it is feasible that radiologists, working in difficult conditions due to the shortage of personnel in the hospital where the present research was conducted, but in the presence of a growing influx of patients with intracranial aneurysms, would allocate little time to the initial analysis of CTA-3D scans as they would be focusing on identifying the presence of a possible aneurysm in patients presenting with a subarachnoid hemorrhage (SAH), which is a neurosurgical emergency.

Therefore, it is plausible that radiologists mostly do not look for possible anatomical variants of the CW during an emergency CTA assessment probably because only a short period of time can be allocated to interpreting scans in the emergency room, as the specialist is under pressure to establish a rapid diagnosis of the intracranial aneurysm in a patient with SAH. Moreover, the PcoAs are quite difficult to visualize on CTA scans, even when these arteries have a normal appearance, due to their diameters being smaller than 1 mm.

## 5. Conclusions

This study, for the first time, compares and correlates the morphological data provided by two methods, i.e., autopsy and antemortem computed tomography–angiography, within the same sample of patients.

Although authors previously considered the occurrence of AcoA aneurysms to only be related to hemodynamic changes induced by nearby arteries, this study identified the involvement of simultaneous anatomical variants of the arteries located in both the anterior and posterior parts of the same circle of Willis in the development and rupture of this aneurysm. Moreover, there was a relationship between the size of AcoA aneurysms and the number of anatomical variants of the circle of Willis.

In addition, this study provides evidence that the analysis of 3D virtual reconstruction CT–angiography images in an emergency setting may not identify anatomical variants associated with AcoA aneurysms, as the time allocated is very limited. However, if sufficient time is devoted to the computed tomography–angiography analysis, anatomical variants of the circle of Willis associated with AcoA aneurysm can be identified with the same degree of accuracy as an invasive postmortem autopsy examination.

The data obtained in this research can help neurologists understand some atypical clinical patterns of stroke and can be useful for neurosurgeons in developing individualized surgical protocols in the case of patients with intracranial aneurysm and choosing the optimal endovascular treatment methods.

## Figures and Tables

**Table 1:** Demographic characteristics of patients with ruptured and unruptured AcoA aneurysms.

Descriptor	Ruptured AcoA Aneurysms (*n* = 14)	Unruptured AcoA Aneurysms (*n* = 2)	Total	*p*-Value
Gender, *n* (%)				0.500 ^†^
Male	8 (57.1%)	2 (100%)	10 (62.5%)	
Female	6 (42.9%)	0 (0.0%)	6 (37.5%)	
Age (mean ± SD), years	63.29 ± 12.89	59.50 ± 13.43	62.81 ± 12.56	0.704 ^‡^
Male	58.50 ± 12.62	59.50 ± 13.43	58.70 ± 12.01	
Female	69.67 ± 11.11	-	69.67 ± 11.11	
Age (years), *n* (%)				1.000 ^†^
≥60	10 (71.4%)	1 (50.0%)	11 (68.8%)	
<60	4 (28.6%)	1 (50.0%)	5 (31.3%)	
Total	14 (100%)	2 (100%)	16 (100%)	

^†^ Chi-squared test (with Fisher correction if necessary); ^‡^ Student’s *t*-test.

**Table 2:** Comparative study of ruptured/unruptured aneurysms according to their external diameters and the number of associated anatomical variants of the arteries constituting circle of Willis.

Descriptor	Ruptured AcoA Aneurysms (*n* = 14)	Unruptured AcoA Aneurysms (*n* = 2)	Total	*p*-Value
External diameters of AcoA aneurysms (mm)	9.50	4.00	8.81	<0.001 ^‡^
No. of associated anatomical variants				0.767 ^†^
1	2 (14.3%)	-	2 (12.5%)	
2	2 (14.3%)	-	2 (12.5%)	
3	7 (50.0%)	1 (50.0%)	8 (50.0%)	
4	3 (21.4%)	1 (50.0%)	4 (25.0%)	

^†^ Chi-squared test (with Fisher correction if necessary); ^‡^ Student’s *t*-test.

**Table 3:** Risks of AcoA aneurysm rupture depending on the diameter of the vessel, and location and type of the associated anatomical variant of the arteries constituting the circle of Willis.

Descriptor	Ruptured AcoA Aneurysms (*n* = 14)	Unruptured AcoA Aneurysms (*n* = 2)	Total	*p*-Value
Size of aneurysm				<0.001 †
<5 mm	-	2 (100.0%)	2 (12.5%)	
5–9.9 mm	8 (57.1%)	-	8 (50.0%)	
≥10 mm	6 (42.9%)	-	6 (37.5%)	
Type of anatomical variant				
Only hypoplastic variant of any artery of the CW	9 (64.3%)	1 (50.0%)	10 (62.5%)	1.000
Only PCA fetal variant	-	-	-	-
Hypoplastic variant of any artery of the CW associated with PCA fetal variant	5 (35.7%)	1 (50.0%)	6 (37.5%)	1.000

^†^ Chi-squared test (with Fisher correction if necessary).

**Table 4:** Correlations between the number of associated anatomical variants of the arteries constituting the circle of Willis and the AcoA aneurysm external diameters.

Descriptor	AcoA Aneurysm External Diameter (Mean ± SD)	*p*-Value
No. of associated anatomical variants		0.409 ^†^
1	6.50 ± 2.12	
2	7.50 ± 0.70	
3	8.63 ± 2.77	
4	11.00 ± 4.83	

^†^ ANOVA test.

**Table 5:** Type of agreement between autopsy data and CT–angiography regarding the presence of AcoA aneurysm and associated anatomical variants of the constituting arteries of the circle of Willis at the initial and postmortem re-analysis.

Type of Agreement	Antemortem Analysis (No. of Cases = 16)	Postmortem Analysis (No. of Cases = 16)	*p*-Value ^†^
Complete agreement	-	16 (100.0%)	<0.001 †
Incomplete agreement	3 (18.8%)	-	
No agreement	13 (81.3%)	-	

^†^ Chi-squared test.

## Data Availability

The data presented in this study are available on request from the corresponding author due to identity restrictions of patient medical records. Before handing in on request, anonymization must be performed.

## Abbreviations

The following abbreviations are used in this manuscript:

ACA	Anterior Cerebral Artery(ies)
AcoA	Anterior Communicating Artery
CW	Circle of Willis
CTA	Computed Tomography–Angiography
ICA	Internal Carotid Artery
PCA	Posterior Cerebral Artery(ies)
PcoA	Posterior Communicating Artery(ies)
SD	Standard Deviation (SD)
